# Effects of Halogen, Chalcogen, Pnicogen, and Tetrel Bonds on IR and NMR Spectra

**DOI:** 10.3390/molecules24152822

**Published:** 2019-08-02

**Authors:** Jia Lu, Steve Scheiner

**Affiliations:** Department of Chemistry and Biochemistry, Utah State University, Logan, UT 84322-0300, USA

**Keywords:** stretching frequency, chemical shielding, atomic charge, NBO

## Abstract

Complexes were formed pairing FX, FHY, FH_2_Z, and FH_3_T (X = Cl, Br, I; Y = S, Se, Te; Z = P, As, Sb; T = Si, Ge, Sn) with NH_3_ in order to form an A⋯N noncovalent bond, where A refers to the central atom. Geometries, energetics, atomic charges, and spectroscopic characteristics of these complexes were evaluated via DFT calculations. In all cases, the A–F bond, which is located opposite the base and is responsible for the σ-hole on the A atom, elongates and its stretching frequency undergoes a shift to the red. This shift varies from 42 to 175 cm^−1^ and is largest for the halogen bonds, followed by chalcogen, tetrel, and then pnicogen. The shift also decreases as the central A atom is enlarged. The NMR chemical shielding of the A atom is increased while that of the F and electron donor N atom are lowered. Unlike the IR frequency shifts, it is the third-row A atoms that undergo the largest change in NMR shielding. The change in shielding of A is highly variable, ranging from negligible for FSnH_3_ all the way up to 1675 ppm for FBr, while those of the F atom lie in the 55–422 ppm range. Although smaller in magnitude, the changes in the N shielding are still easily detectable, between 7 and 27 ppm.

## 1. Introduction

Recent years have witnessed a growing recognition of a range of newly rediscovered noncovalent bonds. Similarly to their closely related H-bond cousin, this class of bonds [1,2,3,4,5,6,7,8,9,10,11,12,13,14,15,16,17,18,19,20,21,22,23,24,25] are derived from a primary electrostatic attraction, supplemented by substantial amounts of charge transfer, polarization, and dispersion ingredients. In addition to their occurrence in small-model dimers, these sorts of interactions are a major factor in the structure and function of much larger supramolecular systems [26,27,28,29,30,31,32,33,34,35].

Rather than utilizing a H atom as a bridge between a pair of molecules, these related interactions incorporate a more electronegative atom, drawn from the right side of the periodic table. The primary difference from the H-bond is that this bridging atom does not have a partial positive charge which can draw in the negative region of an approaching nucleophile. Although the bridging atom may have an overall negative charge, its electrostatic potential is more complex, and quite anisotropic. Taking the R–X bond of halogen atom X as an example, the potential around X is characterized by a negative equator around the R–X bond, accompanied by a positive polar region lying along the extension of the R–X bond. This positive region, commonly referred to as a σ-hole, attracts the nucleophile in the same manner as the H atom within a H-bond.

Depending upon the family of elements from which this bridging atom is drawn, the resulting noncovalent bond is typically dubbed a halogen, chalcogen, pnicogen, or tetrel bond. These interactions share a number of features. Firstly, they are typically of a strength comparable to a H-bond, and are sometimes stronger by a substantial amount. Each such bond is systematically strengthened by electron-withdrawing substituents which intensify the σ-hole. These bonds are usually strengthened as one moves down each column of the periodic table, e.g., Cl < Br < I. First-row atoms, i.e., F, O, N, and C, engage in only weak bonds of this type if at all, but can be coaxed into measurable interactions by appropriate substituents or adding a charge [36,37,38,39,40,41,42]. 

In the case of the H-bond, its spectroscopic consequences have been examined over a span of decades, and are well understood [43,44,45,46]. Such spectra are one of the tools used most often in the toolbox of chemists and biochemists in deciphering the interactions that are present in a complex system. Indeed, the shifts in certain IR spectroscopic bands or NMR peaks are frequently interpreted as a quantitative measure of the strength of each such bond. Work has also unraveled the source of these shifts, in terms of the most fundamental physical characteristics of the molecules involved. The surprise finding that certain H-bonds can shift an IR stretching frequency to the blue, rather than to the red which is the normal situation, has been analyzed and provided new insights into the source of this discrepancy [47,48,49,50,51,52].

However, while a good deal of information has accumulated in recent years concerning certain aspects of the halogen and related bonds, there is less information available concerning their spectra. The data that has appeared [36,53,54,55,56,57,58,59,60,61,62] has been informative, but does not consider these systems in a systematic manner. As such, currently, there is no thorough account of the manner in which each sort of interaction modulates the spectra, nor a solid understanding of the contributing factors. Such information would be crucial in detecting their presence in a given chemical or biological system. It would also be especially useful if correlations could be established, as is already the case with H-bonds, between certain spectroscopic parameters and the strength or geometry of a given bond.

The present work represents an attempt to fill this gap. A number of small-model systems are generated in which a halogen, chalcogen, pnicogen, or tetrel bond is present. Quantum calculations evaluate the strength of each interaction, as well as its geometric properties. The effects of each bond upon the IR and NMR spectra of the system are determined and compared with the binding strength, identity of the bond, and nature of the specific bridging atom. In this way, a systematic set of rules is generated that will assist in identification of these sorts of bonds in a complicated system, and to provide some measure of its strength.

## 2. Methods and Systems

In order to examine this question systematically, three different Lewis acids were considered for each type of bond. The central atom is drawn from the second, third, and fourth row of the periodic table, where these bonds are the strongest. An F atom was placed as a substituent on each such atom, as its electron-withdrawing power will facilitate the development of the noncovalent bond of interest. Thus, FCl, FBr, and FI were taken as prototype halogen bonding molecules. In a related manner, chalcogen bonds were considered via FSH, FSeH, and FTeH. The corresponding pnicogen and tetrel-bonding units are, respectively, (FPH_2_, FAsH_2_, FSbH_2_) and (FSiH_3_, FGeH_3_, FSnH_3_). NH_3_ was taken as the common electron donor/nucleophile due, first, to its strength as a base. As a second benefit, its small size facilitates analysis and avoids complicating secondary interactions.

Calculations were carried out with the M06-2X variant [63] of DFT within the framework of the Gaussian 09 [64] suite of programs. The aug-cc-pVDZ basis set [65,66] was used for all atoms except fourth row I, Te, Sb and Sn, for which relativistic effects were incorporated via the aug-cc-pVDZ-PP pseudopotential [67,68]. The reliability of such methods applied in similar systems is supported by numerous previous works [69,70,71,72,73,74,75]. Geometries were optimized and harmonic frequency analysis assured the presence of a minimum.

Interaction energies, E_int_, were taken as a measure of the strength of each interaction. This quantity refers to the difference in energy between the fully optimized complex and the energy sum of the two monomers, both in the geometry that pertains to the complex. This quantity was corrected for basis set superposition [76] by the counterpoise prescription [77], originally proposed by Boys and Bernardi. NMR chemical shielding was assessed via the GIAO procedure [78,79] incorporated within Gaussian. The Natural Bond Orbital (NBO) method [80] was utilized to extract natural atomic charges using the NBO-3.1 program, included within the Gaussian-09 program.

## 3. Results

**Figure 1:**
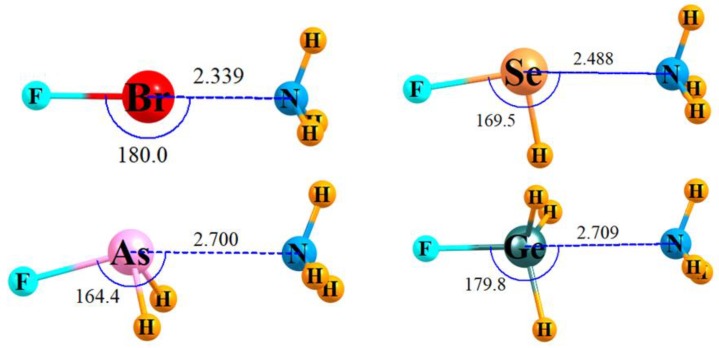
Geometries of sample noncovalently bonded complexes. Distance in Å and angles in degrees. The geometry that includes FSeH is not a pure minimum as it includes one negative frequency, which corresponds to torsional rotation of the NH_3_ group.

The optimized geometries of several sample complexes are pictured in Figure 1. The others are quite similar and are contained in Figure S1. The NH_3_ approaches the central A atom of each molecule opposite to the A–F bond (where A denotes the halogen, chalcogen, etc., atom) although in the case of the chalcogen and pnicogen bonds, the θ(FA⋯N) angle is not quite 180° due to considerations of the orientation of the σ-hole and secondary interactions [81,82,83,84]. It is the A–F bond of the Lewis acid molecule which ought to be most susceptible to modifications due to the interaction with the base, and where focus is placed below.

### 3.1. Energetics and IR Spectra

The first column of Table 1 displays the interaction energy of each complex. It may be noted that these noncovalent bonds are all rather strong. To place this result in context, the water dimer, as a H-bonding parallel, has an interaction energy on the order of 5 kcal/mol [85,86]. The quantities in Table 1 are all larger than this amount, and in some cases are as much as four times larger.

As is typically the case, each sort of interaction strengthens as the bridging atom, whether halogen, chalcogen, or otherwise, grows larger (with one minor exception for Ge vs. Si). In terms of the class of the bond, the halogen bonds are the strongest, followed by chalcogen, tetrel, and pnicogen. A common measure of the charge transfer taking place in each noncovalent bond derives from the NBO second-order perturbation energy E(2). In each case, the donor is the N lone pair and the acceptor is the σ*(A–F) antibonding orbital. These quantities in the second column of Table 1 generally follow the interaction energy trends, albeit imperfectly. For example, it is the second-row tetrel atom Si which shows a larger value of E(2) than either third- or fourth-row analogs. Third-row Br is associated with the largest E(2) of the three halogen atoms. 

The transfer of electron density into the antibonding σ*(A–F) bond ought to weaken and lengthen this bond, which can be seen by the stretches, Δr, documented in the third column of Table 1. As in the prior parameters, Δr follows the general pattern halogen > chalcogen > tetrel ~ pnicogen. This A–F bond weakening is also manifested in a red shift of the stretching frequency Δν. Like the energetics, the halogen > chalcogen > tetrel > pnicogen order also holds here. However, there is also a very significant difference. Unlike the energetics, these red shifts are largest for the lightest of each class of A atom. Taking the halogens as an example, this shift of 175 cm^−1^ for Cl drops down to 107 for Br and then to 49 for I. However, perhaps most importantly, these shifts are all large enough that they ought to be plainly evident in the IR spectra of these systems, falling in the range of 42–175 cm^−1^. 

As is typical of H-bonds, the red shift is accompanied by an intensification of the stretching band. The magnification of this intensity is displayed in the last column of Table 1 as the ratio of intensities in the complex and the monomer. This intensification ratio varies from as small as 1.2, up to 7.7 for FCl⋯NH_3_. This quantity decreases smoothly as each A atom becomes larger, and also follows the general pattern halogen > chalcogen > tetrel > pnicogen, which is very much in line with the red shifts, Δν.

### 3.2. NMR Shielding

The NMR chemical shielding of each of the salient atoms is reported in Table 2 as a change from the shielding within the uncomplexed monomer. While the shielding change in the central A atom is positive, its magnitude is highly variable. The halogen systems are a case in point. The shielding change on the I atom is 72 ppm, which rises to 478 ppm for Cl and then is dramatically larger at 1675 ppm for Br. Indeed, there does appear to be a pattern, in which the third-row atom in each class undergoes the largest change and the fourth-row atom, the smallest. The order observed here is fairly consistent with those in Table 1, with halogen the largest, followed by chalcogen. However, the pnicogen bonds clearly display larger NMR shifts than the tetrel atoms.

Unlike the increased shielding of the A atoms, the F substituents undergo a dramatic decrease, in the range between 55 and 422 ppm. However, the order is the same: halogen > chalcogen > pnicogen > tetrel. On the other hand, the dependence upon the particular row of the periodic table is erratic. Δσ clearly increases in magnitude as the halogen atom grows in size, but is less sensitive for the three other types of bonds.

Since the H atoms on the Lewis acid are not directly involved in the noncovalent bond, one would not anticipate their shielding to change much upon complexation. Nevertheless, while certainly smaller, these changes ought to be detectable. The H shielding increases in all cases: it is largest for the chalcogen bonds in the 4–5 ppm range, drops to about 1 ppm for pnicogen, and then below 0.5 ppm for the tetrel bonds.

As the electron donor atom, it is not surprising that the chemical shielding of the N atom of NH_3_ suffers a drop upon complexation. This decrease falls in the 7–27 ppm range so again, ought to be easily detectable. Unlike the other parameters discussed above, it is the tetrel bond that displays the largest perturbation for the N shielding change. The halogen bonds are somewhat less sensitive, followed by the two others. There is no clear pattern in terms of periodic table row. For example, while it is the lightest halogen atom that changes the N shielding the most, it is the heaviest of the pnicogen atoms that has this distinction.

### 3.3. Atomic Charges

One would expect that the shielding of each atom ought to have some relation with the total charge surrounding that atom. The changes in the atomic charge of each atom that accompany the complexation are thus displayed in Table 3. The negative values for the A atoms indicate an increased surrounding electron density, which is consistent with the greater shielding indicated in Table 2. On the other hand, the magnitudes of the charge changes bear little resemblance to the shielding. For example, the P, As, and Sb atoms all increase their negative charge by the same 0.035 e, yet there is a great variability in the shielding change, from 5 ppm for Sb to as high as 166 ppm for As. As an even greater disparity, the F atoms also accrue additional density during the complexation, comparable to that observed in A, but suffer a very substantial drop in their shielding, opposite to the increase in shielding in A. 

The changes on the H atoms are small, 0.005 e or less, but vary in sign. While positive for the chalcogen and pnicogen bonds, they reverse sign for the tetrel bonds, even though the shielding change is positive for all systems. With respect to electron donor N, it suffers a loss of density, with its atomic charge growing more positive. However, this change is not universal, and is even negative for a few cases. There is some similarity to Δσ in that the most positive charge changes occur in the halogen bonds, which also display the largest change in N shielding. Both quantities are smaller for the chalcogen bonds. However, the small N-charge changes in the tetrel bonds are in dissonance with their large shielding changes.

### 3.4. Electron Density Shifts

**Figure 2:**
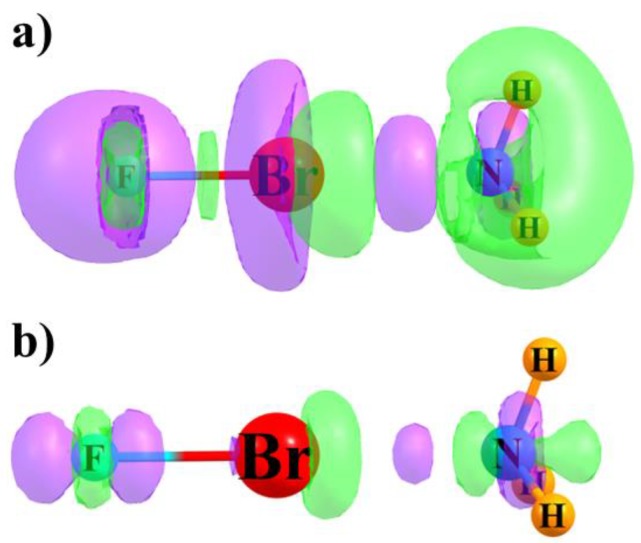
Density shifts caused by complexation between FBr and NH_3_. Purple areas indicate gains and losses are shown in green. Contours shown represent (**a**) 0.001 au and (**b**) 0.005 au.

A more thorough examination of the electron density shifts can perhaps offer some deeper insights into the changes in both atomic charges and NMR chemical shielding perturbations. Figure 2 displays the shifts in density caused by the complexation of NH_3_ with FBr as an example. The purple regions represent gains of density and losses are shown in green. Two different levels are shown to provide a more complete picture. The Δρ = 0.001 au contour is displayed in Figure 2a, while the larger value of 0.005 au in Figure 2b focuses attention on regions of more concentrated charge gain and depletion.

Considering the central Br atom first, in Figure 2a, a purple region of density gain to its left and a green area of density loss to its right are apparent. The former is larger than the latter which accounts for the more negative charge on the Br atom, as shown in Table 3. Consideration of only the 0.005 au contour in Figure 2b would offer a contrasting picture as the green area is much more extensive than the purple. The increased density around the Br atom is in part responsible for the higher NMR shielding indicated in Table 2. In the case of the N atom of NH_3_, the green lobes of density loss appear to dominate at either contour value, which is consistent with the more positive charge of N in Table 3 and its reduced shielding in Table 2.

The F atom offers an interesting picture. One can see a green region of density loss close to the nucleus in Figure 2a, which is surrounded on both sides by two more extensive purple lobes of density gain. These areas persist in the more concentrated density loss regions shown in Figure 2b. As indicated in Table 3, it is the purple areas of gain that are more influential, as the overall charge on the F atom becomes more negative. On the other hand, the shielding of this atom is reduced substantially. One way of viewing this apparent paradox is consideration of the placement of these lobes. The green region of density loss occurs directly around the F nucleus, where this may have a more direct influence on the shielding than the more distant purple regions of density gain.

The density shifts of the FBr⋯NH_3_ complex are not unique, but rather characteristic of all of the systems examined here. Comparable density shift diagrams are provided in Figure S2, where the similarities are evident.

## 4. Discussion and Conclusions

Prior studies have provided confidence that NMR and IR data computed by DFT and ab initio approaches offer some reliability [38,58,87,88,89,90]. As a specific example, an early study [91] calculated nuclear shielding changes involved in the P⋯N pnicogen bond in the FH_2_P⋯NH_3_ complex at the MP2/aug’-cc-pVTZ level. The P and N atoms changed their shielding by +77 and −10 ppm, respectively, which is similar to the quantities described here.

There have been several studies which have attempted to relate spectroscopic data with noncovalent bond energies. Mokrai et al. observed [92] through-space coupling constants within a pnicogen bond in solid-state NMR spectra. The NMR chemical shift of the C that is covalently attached to the halogen bonding I in a C–I bond increases markedly as the XB is enhanced by an anionic electron donor [93]. Within the framework of C–tetrel bonds, Southern and Bryce [38] observed a strong correlation between the C chemical shifts and the length of the C–tetrel bond, although this correlation deteriorated when compared to the actual strength of the bond. One should be cautious, however, in drawing parallels between observations in the broad context of noncovalent bonds in general and a limited set of tetrel bonds involving only the C atom, which tends to form weak tetrel bonds. As another point of agreement, the N electron donor of an aromatic ring suffers a decrease in its chemical shielding by a variable amount within a CX⋯N halogen bond [94], up to as much as 19 ppm. As in the case of our NH_3_ electron donor, halogen bonding causes deshielding of the F atom when it acts as an electron donor in a halogen bond [95]. This is also consonant with our own findings, a red shift of the F–X stretching frequency has been observed [96] in its halogen bonds to aromatic N electron donors, in the range of about 140 cm^−1^.

In summary, the noncovalent bonds that fall into the categories of halogen, chalcogen, pnicogen, and tetrel bonds have a number of spectroscopic features in common. As the base takes a position opposite the F–A bond of the FH_n_A molecule, the covalent A–F bond is weakened and stretched by 0.025–0.060 Å. The stretching frequency of this bond is shifted to the red by at least 40 cm^−1^, up to as much as 175 cm^−1^. The amount of this red shift is largest for the A atoms of the second row of the periodic table: Cl, S, P, and Si. Halogen bonds cause the largest shift, followed by chalcogen, tetrel, and then pnicogen.

Each of the atoms involved in these bonds undergoes a characteristic change in its NMR signal. The shielding of the A atom is increased, while that of the F and electron donor N atom are lowered. The changes observed for the A and F atoms follow the order halogen > chalcogen > pnicogen > tetrel, but in the case of the N atom, the order changes to tetrel > halogen > chalcogen ~ pnicogen. Unlike the IR frequency shifts, it is the third-row A atoms that undergo the largest change in NMR shielding and the fourth row, the smallest. The latter small quantity contrasts with the fact that the interaction energies between the noncovalent bonds involving these heavy atoms are the largest. In terms of magnitude, Δσ for A is highly variable, ranging from negligible for FSnH_3_ all the way up to 1675 ppm for FBr. F shielding changes are not quite as variable, covering the range from 55 to 422 ppm. Although smaller in magnitude, the changes in the N shielding are still easily detectable, between 7 and 27 ppm.

## Figures and Tables

**Table 1:** Interaction energy, NBO charge transfer energy, bond length change, and bond stretch frequency and intensity change upon forming complex with NH_3_.

	−E_int_, kcal/mol	E(2), kcal/mol	Δr (AF), Å	Δν (AF), cm^−1^	I Ratio ^a^
halogen					
FCl	12.01	39.3	0.060	−174.9	7.7
FBr	16.28	51.3	0.061	−107.1	5.0
FI	19.17	43.6	0.055	−48.6	2.9
chalcogen					
FSH	9.12	21.0	0.034	−85.8	3.0
FSeH	12.25	30.9	0.042	−70.3	2.6
FTeH	16.14	20.9 ^b^	0.042	−44.8	1.9
pnicogen					
FPH_2_	7.39	13.9	0.022	−58.1	1.9
FAsH_2_	8.98	16.3	0.029	−47.3	1.8
FSbH_2_	11.75	17.2	0.034	−42.1	1.5
tetrel					
FSiH_3_	9.01	18.1	0.027	−74.7	3.4
FGeH_3_	8.50	12.7	0.025	−55.2	1.6
FSnH_3_	12.18	13.3	0.030	−54.9	1.2

^a^ I_complex_/I_monomer_ for AF stretch, ^b^ σ(Te-N) → σ*(Te-F)—NBO treats a complex as a single unit.

**Table 2:** Changes in chemical shielding (ppm) that accompany complexation with NH_3_.

	A	F	H ^a^	N
halogen				
FCl	478.2	−272.7	-	−20.2
FBr	1674.9	−391.2	-	−17.6
FI	72.3	−421.7	-	−6.9
chalcogen				
FSH	264.0	−184.1	3.8	−11.5
FSeH ^a^	847.5	−234.0	4.9	−12.5
FTeH	34.8	−233.4	5.0	−8.3
pnicogen				
FPH_2_	68.2	−92.0	1.2	−10.6
FAsH_2_	165.9	−88.6	1.1	−8.7
FSbH_2_	5.0	−88.3	1.0	−12.4
tetrel				
FSiH_3_	46.3	−70.0	0.4	−26.6
FGeH_3_	70.7	−54.8	0.3	−16.3
FSnH_3_	−0.3	−59.7	0.3	−21.3

^a^ average of all H atoms on the central atom.

**Table 3:** Changes in natural atomic charge (e) that accompany complexation with NH_3_.

	A	F	H ^a^	N
halogen				
FCl	−0.076	−0.087	-	0.067
FBr	−0.084	−0.099	-	0.066
FI	−0.067	−0.086	-	0.031
chalcogen				
FSH	−0.044	−0.050	0.005	0.019
FSeH ^a^	−0.058	−0.061	0.004	0.026
FTeH	−0.053	−0.056	0.001	0.007
pnicogen				
FPH_2_	−0.035	−0.029	0.003	0.003
FAsH_2_	−0.035	−0.034	0.002	0.001
FSbH_2_	−0.036	−0.035	−0.001	−0.007
tetrel				
FSiH_3_	−0.040	−0.028	−0.005	0.005
FGeH_3_	−0.016	−0.027	−0.005	−0.004
FSnH_3_	−0.012	−0.029	−0.010	−0.011

^a^ average of all H atoms on central atom.

## Supplementary Materials

The following are available online at https://www.mdpi.com/1420-3049/24/15/2822/s1, Figure S1: Optimized geometry of complexes with NH_3_; Figure S2: Electron density shifts caused by complexation between Lewis acid and NH_3_.
