# Supplementary material for: Effects of Halogen, Chalcogen, Pnicogen, and Tetrel Bonds on IR and NMR Spectra
Source: Molecules. 2019 Aug 2;24(15):2822. doi: 10.3390/molecules24152822 (PMC6696224; doi:10.3390/molecules24152822)
Supplement: Supplementary file 1 [file molecules-24-02822-s001.pdf]

SUPPLEMENTARY INFORMATION

Effects of Halogen, Chalcogen, Pnicogen, and Tetrel Bonds on IR and NMR Spectra

Jia Lu and Steve Scheiner*

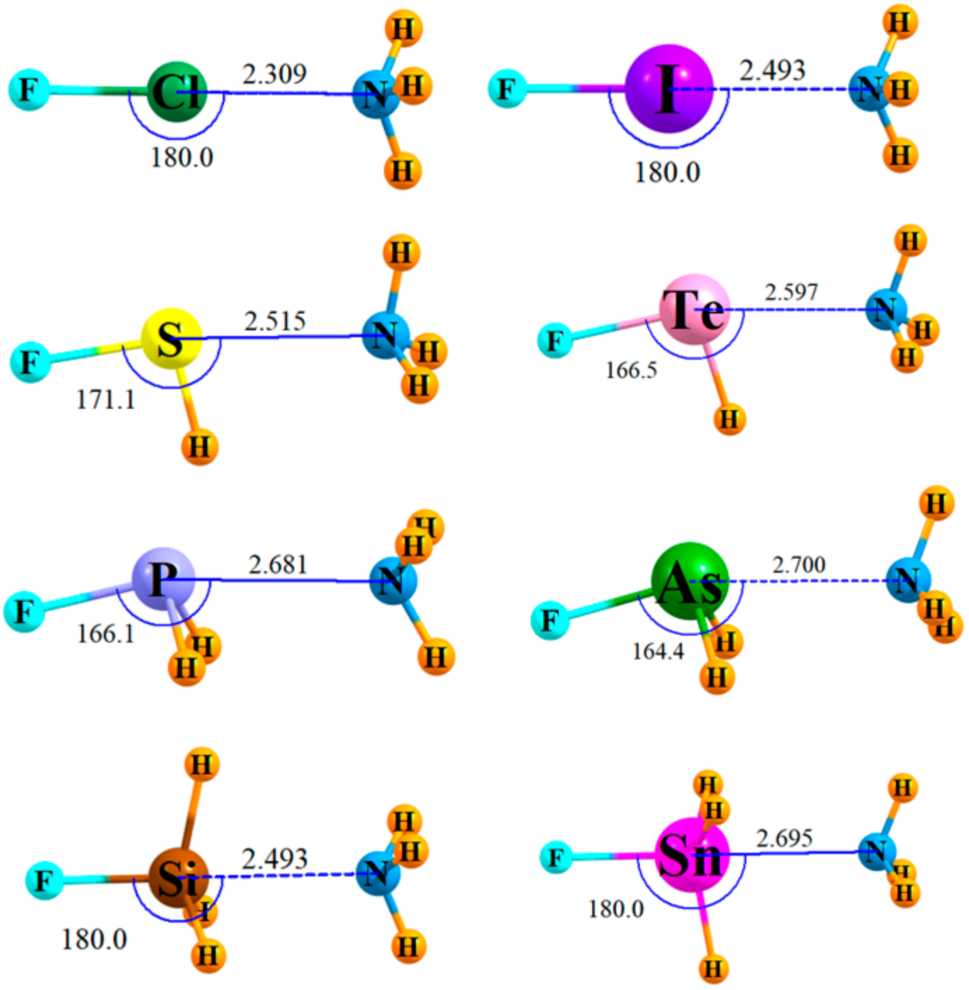

Fig S1. Optimized geometry of complexes with NH_3. The geometry that includes FSH is not a pure minimum as it includes one negative frequency which corresponds to torsional rotation of the NH_3 group.

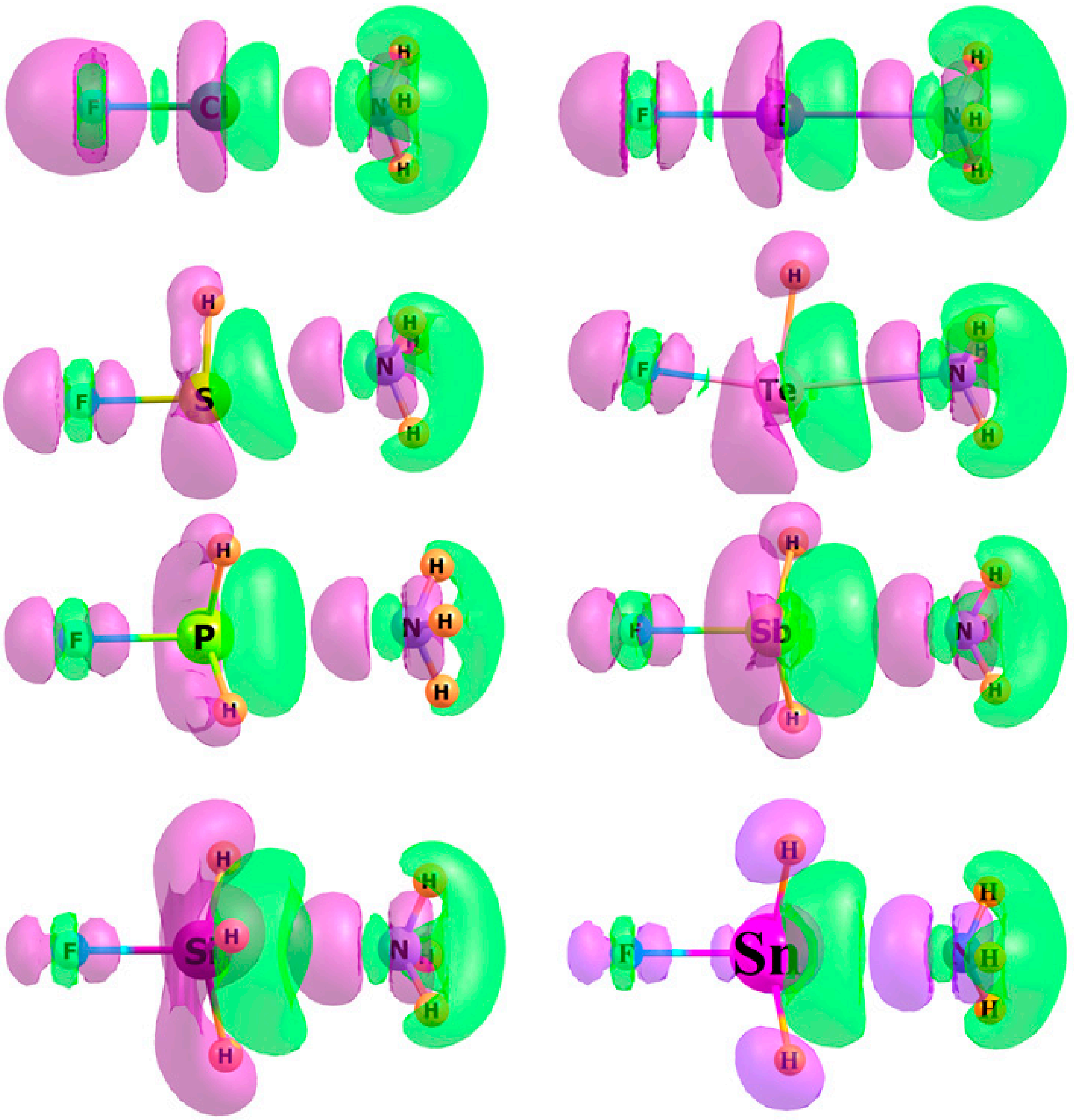

Fig S2. Electron density shifts caused by complexation between Lewis acid and NH_3. Purple areas indicate gains and losses are shown in green. Contours shown represent 0.001 au.
